# Significance of pharmacist intervention to oral antithrombotic therapy in the pharmaceutical outpatient clinic of cardiovascular internal medicine: a retrospective cohort study

**DOI:** 10.1186/s40780-023-00296-9

**Published:** 2023-09-05

**Authors:** Tomoko Kurimura, Kazuhiro Yamamoto, Hidekazu Tanaka, Takayoshi Toba, Takeshi Kimura, Yasushi Habu, Kotaro Itohara, Yumi Kitahiro, Tomohiro Omura, Ikuko Yano

**Affiliations:** 1 Department of Pharmacy, Kobe University Hospital, Kobe, Japan; 2 Division of Cardiovascular Medicine, Department of Internal Medicine, Kobe University Graduate School of Medicine, Kobe, Japan; 3 Department of Drug Informatics, Kobe Pharmaceutical University, Kobe, Japan

**Keywords:** Pharmacist intervention, Antithrombotic drugs, BARC bleeding, Outpatient

## Abstract

**Background:**

Optimised antithrombotic therapy requires clinical experience and an understanding of the current guidelines. This retrospective study aimed to evaluate whether pharmacist interviews and interventions with patients taking oral antithrombotic drugs in the pharmaceutical outpatient cardiology clinic had favourable clinical outcomes including decreased bleeding.

**Methods:**

The participants included patients visiting the outpatient clinic of cardiovascular internal medicine at the Kobe University Hospital from January–December 2017, and were taking oral antithrombotic medication. The observation period was from the first visit to the outpatient clinic to October 2021 or death. Patients who received pharmacist intervention more than twice were defined as the pharmacist intervention group. Two control patients per one pharmacist intervention group individual were selected from the non-intervention pool matched for age, gender and antithrombotic medication type.

**Results:**

Of the 895 eligible patients, 132 were in the pharmacist intervention group and 264 were selected for the matched non-intervention group. Bleeding events according to the Bleeding Academic Research Consortium criteria over type 2 were significantly lower in the pharmacist intervention group compared with the non-intervention group (17.4% *versus* 28.4%, *P* = 0.019). There were no significant differences in mortality and heart failure hospitalisation frequency, stroke, or cardiovascular events between the groups. Multivariate analysis identified age (≥ 65 years) and pharmacist intervention as factors associated with bleeding (odds ratio = 2.29 and 0.51, respectively).

**Conclusion:**

Pharmacist intervention in the outpatient clinic of cardiovascular internal medicine was effective in reducing the risk of bleeding in patients undergoing antithrombotic therapy.

## Background

Antithrombotic therapy, including anticoagulant and antiplatelet therapy, is an important therapeutic strategy for patients with ischemic cardiac disease and atrial fibrillation (AF). Antithrombotic drugs must be carefully used for their selection and dose adjustment to obtain the optimal anticoagulation and antiplatelet effects [1, 2]. These drug doses are adjusted considering the balance between the risk of bleeding and preventive effects on thrombotic events. In recent years, the benefits of shortening the duration of concomitant antithrombotic drug use and reduction in the number of concomitant antithrombotic drugs has been suggested [3–5]. Regarding the treatment period and reduction in the number of antiplatelet drugs after percutaneous coronary intervention (PCI), the WOEST study showed that the dual antiplatelet therapy (DAPT) group had significantly lower bleeding and stent thrombosis incidents than the triple antithrombotic drug group [3]. The major relevant societies in the world suggest that the usage duration of the three antithrombotic drugs should be shortened to no more than two weeks [6–8]. Additionally, the duration of the concomitant use of two antithrombotic drugs was recommended for 12 months, followed by use of direct oral anticoagulant (DOAC) alone. The duration of DAPT has also been shortened to 1–3 months in cases with a lower risk of thrombosis [6–8]. Along with these recommendations, the appropriate use of DOACs, warfarin and other antithrombotic drugs has become a crucial concern. For the prognosis of patients with AF, the physicians and pharmacists should be familiar with the latest revised guidelines of these complicated medications and change the individual pharmacotherapy accordingly.

On one hand, pharmacist interventions for outpatient pharmacotherapy have been shown to improve the clinical outcomes for patients with a variety of diseases. For cancer outpatients, providing drug information to physicians and patients as well as performing patient counselling by pharmacists decreased adverse events such as nausea and pain [9]. For outpatients with type 2 diabetes, the intervention by pharmacists in the diabetes care team improved the glycated haemoglobin A1c (HbA1c) levels, blood sugar levels, blood pressure, cholesterol levels, triglyceride levels, cardiovascular event risk and patient adherence [10]. In patients with chronic kidney disease, pharmacist interventions reduced the number of medications, hospitalisation rate and duration of hospitalisation [11]. However, these reports were based on evaluating soft outcomes resulting from pharmacist intervention, and hard outcomes over the long-term resulting from pharmacist intervention in outpatient cardiovascular clinics has not fully been reported.

This retrospective study aimed to evaluate the effectiveness of pharmacist intervention for outpatients taking antithrombotic drugs associated with heart diseases such as ischemic heart disease and AF on the clinical outcomes, such as improved bleeding risk, thrombotic risk and heart failure prognosis.

## Methods

### Study design

This was a single-center retrospective matched cohort study with four-year observation period.

### Procedure of pharmacist intervention with outpatients

**Fig. 1:**
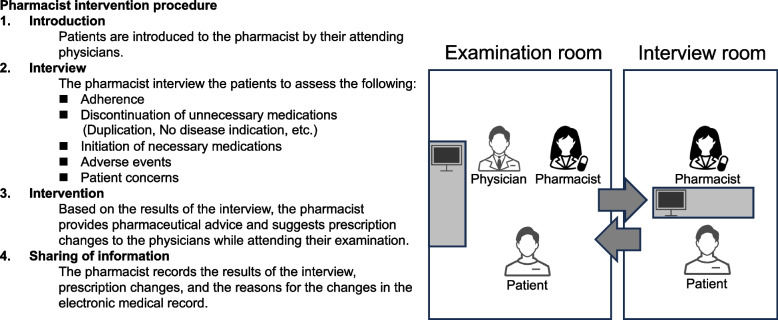
Diagram detailing the pharmacist intervention procedure as mentioned in the previous report [12]

Pharmacist interventions with outpatients were conducted according to the procedures described in the previous report (Fig. 1) [12]. The pharmacist intervention was targeted to all patients who received medical examinations by the specific physician in the specific time slot. The specific physician in the specific examination time slot regularly changed within a year, according to the rotation of the department of cardiovascular internal medicine. The pharmacist attended the medical examination for outpatients. Patients were interviewed by the pharmacist in a separate room before and after the examination as necessary. Each patient was interviewed at every time of the outpatient clinic visit. Pharmacist interventions consisted of checking all prescription drugs from the hospital and other healthcare facilities, laboratory test values, and physical assessments by the procedures described in the previous reports [12, 13], adherence and adverse events. Based on the latest guidelines, reports and labels of each drug, prescription revisions were suggested by the pharmacist to the physician. Any changes in the prescription, such as discontinuation or dose adjustment of the medications, were decided with the consent of the patient and attending physician. Pharmacist intervention in this study was entirely carried out by only one pharmacist who has been working at the hospital for 15 years as of 2017 and who was a Board certified Pharmacotherapy Specialist.

### Patients

The participants were patients who visited the outpatient clinic of cardiovascular internal medicine at the Kobe University Hospital from January to December 2017 and were taking antithrombotic drugs. Antithrombotic drugs were defined as anticoagulant drugs, such as DOAC and warfarin, and antiplatelet drugs such as aspirin and thienopyridines. The four physicians specialising in cardiovascular internal medicine collaborated with the pharmacist intervention, and there was no patient selection bias with regards to disease or prescription drug in the two groups. The observation period was from the first visit during the registration period to October 2021 or the date of death. Patients who received pharmacist interventions more than twice during the observation period were included in the intervention group. The patients who never receive pharmacist intervention during the observation period were included in the non-intervention group as controls. Due to a large number of patients in the non-intervention group, the non-intervention group was selected to be demographically matching with the aim of achieving sample balance. The steps of the matching process were as follows: In the first step, for each patient in the intervention group, patients whose age fell within the range of ± 5 years were selected from the non-intervention group. In the second step, patients whose gender matched each patient in the intervention group were selected from patients selected in the first step. In the final step, two patients matched for the type of antithrombotic drugs that they were taking were selected from patients selected in the second step. If there was an applicable patient in the non-intervention group who was taking the same antithrombotic drug as the intervention group, this patient was selected as priority. If no patient who took the same drug was applicable, patients who took antithrombotic drugs in the same category (anticoagulant drugs or antiplatelet drugs) were selected as priority. If no patients met the category of antithrombotic drugs found in the non-intervention group, the matching step for antithrombotic drugs was omitted.

### Data collection

All data were collected retrospectively from the electronic medical records. Patient backgrounds (gender, age, serum creatinine, estimated glomerular filtration rate (eGFR), aspartate aminotransferase (AST), alanine aminotransferase, indication for anticoagulation, current antithrombotic drug and history of PCI, coronary artery bypass grafting (CABG) and stroke), symptoms for bleeding and the incidence of stroke, coronary events, heart failure, death and hospitalisation caused by heart failure were collected. The events that occurred outside of the hospital after the hospital visit were evaluated by the reports from the external institutions in each patient’s medical record.

### Outcomes

The primary outcome was bleeding greater than type 2 as defined by Bleeding Academic Research Consortium (BARC) [14]. Secondary outcomes were death from any cause, stroke, cardiovascular events and hospitalisation for heart failure. The incidence of cardiovascular events was defined as receiving a PCI or CABG for myocardial infarction or unstable angina. These outcomes were compared between the patients in the intervention and non-intervention groups.

### Statistical analysis

Continuous variables were summarised as mean ± standard deviation (SD) and compared using the Student’s *t*-test. Categorical variables were summarised as proportions and compared using Fisher’s exact test. The cumulative incidence rate of BARC bleeding greater than type 2 was estimated using the Kaplan–Meier method, and significant differences were determined using the log-rank test. The initiation of the analysis was defined as a first visit during the observation period. The data for those who completed the follow-up period or died before a bleeding event were censored.

Logistic regression analysis was performed to examine the risk factors associated with bleeding defined by BARC as greater than type 2 and to estimate the odds ratio (OR) and 95% confidence interval (CI). Explanatory variables with known factors were defined as abnormal renal/liver function, elderly and drugs concomitant use, which are included in the HAS-BLED score. Abnormal renal function was defined by two patterns to broadly evaluate the impact of reduced renal function on antithrombotic drugs-induced bleeding; a serum creatinine level over 2.26 mg/dL in accordance with the original HAS-BLED score and an eGFR less than 60 mL/min/1.73m^2^. Labile international normalised ratio (INR), which is also included in the HAS-BLED score, was not evaluated because it is a laboratory test specific to warfarin use. Unknown bleeding factors were evaluated in the multivariate analysis, because the usefulness of the HAS-BLED score in patients with ischemic cardiac disease and deep venous thrombophlebitis included in this study was not established. Therefore, the multivariate logistic regression analysis was performed using the forced entry procedure with the variables included with a *P*-value of < 0.10 in the univariate analysis as unknown factors in addition to the HAS-BLED score as well-known factors.

The sample size is not calculated based on the prior hypothesis, because this study was required to include all patients who received pharmacist interventions during the period. The number of patients who received pharmacist interventions during the period was estimated as 200 patients. The previous study revealed that the frequency of bleeding defined by BARC as type 2 and 3 was 14.3% (effect size: 55%) and 31.7% in patients who received double antithrombotic therapy and triple antithrombotic therapy, respectively [3]. If these reported values were reproduced in the pharmacist intervention group and the non-intervention group in this study, the statistical power was 99.8%. In the case that the effect size was conservatively assumed to be about 40%, the statistical power was 88.0%.

A *P*-value < 0.05 (two-sided) was considered statistically significant. All statistical analyses were conducted using R 4.0.3 software.

## Results

### Patients

**Fig. 2:**
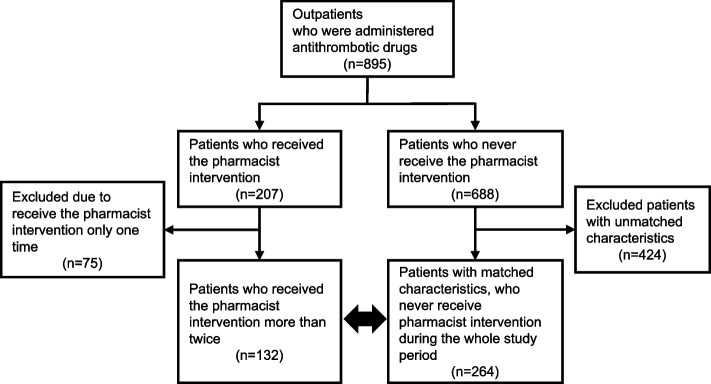
Flow diagram showing the patients targeted for the study

A flowchart of patient selection is shown in Fig. 2. In 2017, 895 patients visited the cardiology department as outpatients and were taking antithrombotic drugs. Of these patients, 132 patients received pharmacist intervention more than twice, and there were 75 patients who received pharmacist intervention only once and were excluded from the study. The patient backgrounds are shown in Table 1. The observation period in the intervention group was significantly shorter than those in the non-intervention group. The median of intervention periods was 224 days (27–1742 days), and there were 43 patients (32.6% of the intervention group) who received the pharmacist intervention for more than 3 years. The median number of interventions per patient was 5.5 (2–31). The ratio of deep vein thrombosis as an indication for anticoagulation in the intervention group was significantly lower than those in the non-intervention group. The type of DOAC at the start of the observation was significantly different between the intervention and non-intervention groups. Other characteristics except AST were not significantly different between the intervention and non-intervention groups. As the information of medical staff involved in the outpatient clinics, the median years (minimum–maximum) of physicians’ experience were 17 years (11–25 years) in the intervention group and 18 years (8–30 years) in the non-intervention group. Additionally, one certified nurse for chronic heart failure nursing was involved in outpatient care across the intervention and non-intervention groups.

**Table 1:** Patient characteristics

	Intervention group (*n* = 132)	Non-intervention group (*n* = 264)	*P-*values
Observation period (day), Mean ± SD	1638 ± 81	1702 ± 50	< 0.001
Intervention period (day)^a^, Median (range)	224 (27–1742)	-	-
Number of interventions, Median (range)	5.5 (2–31)	-	-
Age (year), Mean ± SD	73.2 ± 12.2	72.6 ± 11.8	0.62
Male	94 (71.2%)	188 (71.2%)	1.00
History of stroke (yes)	20 (15.1%)	37 (14.0%)	0.76
History of PCI (yes)	43 (32.5%)	102 (38.6%)	0.27
History of CABG (yes)	8 (6.0%)	25 (9.6%)	0.34
Laboratory data			
SCr (mg/dL), Mean ± SD	1.09 ± 0.48	1.06 ± 0.54	0.66
eGFR (mL/min/1.73m^2^), Mean ± SD	55.7 ± 19.4	57.2 ± 18.4	0.48
AST (U/L), Mean ± SD	29.4 ± 18.6	25.6 ± 11.5	0.011
ALT(U/L), Mean ± SD	25.7 ± 24.7	23.2 ± 16.3	0.23
Indication for anticoagulation			
Atrial fibrillation	65 (49.2%)	108 (40.9%)	0.13
Ischemic heart disease	53 (40.1%)	116 (43.9%)	0.52
Deep vein thrombosis	7 (5.3%)	33 (12.5%)	0.033
Antithrombotic drug			
Warfarin	51 (38.6%)	103 (39.0%)	1.00
DOAC	34 (25.7%)	53 (20.0%)	0.20
Apixaban	22 (16.7%)	23 (8.7%)	0.028
Rivaroxaban	6 (4.5%)	16 (6.1%)	0.65
Dabigatran	1 (0.8%)	14 (5.3%)	0.025
Edoxaban	5 (3.8%)	0	0.004
Antiplatelet drug	63 (47%)	141 (53.4%)	0.29
Aspirin	55 (41.7%)	132 (50.0%)	0.14
Clopidogrel	11 (8.3%)	29 (11.0%)	0.48
Prasugrel	13 (9.8%)	16 (6.1%)	0.22
Cilostazole	3 (2.3%)	4 (1.5%)	0.69
Ticlopidine	1 (0.8%)	3 (1.1%)	1.00
Sarpogrelate	0	3 (1.1%)	0.55

*SD* Standard deviation, *PCI* Percutaneous coronary intervention, *CABG* Coronary artery bypass grafting, *DOAC* Direct oral anticoagulant

^a^Intervention period was the duration from the first to the last visit with pharmacist intervention

### Event incidence rate

Table 2 shows the event incidences in the pharmacist intervention and non-intervention groups. The incidence rates of BARC bleeding greater than type 2 were 17.4% (23/132 patients) in the pharmacist intervention group and 28.4% (75/264 patients) in the non-intervention group, and the pharmacist intervention group had a significantly lower incidence rate (*P* = 0.019). Death was confirmed in 13.6% and 9.0% of the pharmacist intervention and non-intervention groups, respectively (*P* = 0.17). The rate of stroke events was 3.0% and 4.9% in the pharmacist intervention and non-intervention groups, respectively (*P* = 0.44). Cardiovascular events occurred at 3.8% and 9.1% in the pharmacist intervention and non-intervention groups, respectively (*P* = 0.066). Hospitalisation due to heart failure was observed at 15.1% and 14.8% in the pharmacist intervention and non-intervention groups, respectively (*P* = 1.00).

**Table 2:** Comparison of event incidences in the pharmacist intervention and non-intervention groups

	Intervention group (*n* = 132)	Non-intervention group (*n* = 264)	*P-*values
BARC bleeding greater than type 2	23 (17.4%)	75 (28.4%)	0.019
Death	18 (13.6%)	24 (9.0%)	0.17
Stroke events	4 (3.0%)	13 (4.9%)	0.44
Cardiovascular events^a^	5 (3.8%)	24 (9.1%)	0.066
Hospitalizations due to heart failure	20 (15.1%)	39 (14.8%)	1.00

^a^Cardiovascular events are defined as undergoing PCI or CABG for myocardial infarction or unstable angina

### Evaluation of the bleeding risk factors

Table 3 shows the results of the logistic regression analysis according to BARC bleeding defined as BARC greater than type 2. Univariate analysis showed a significant association of bleeding with elderly (over 65 years old), eGFR (less than 60 mL/min/1.73m^2^) and pharmacist intervention. In the multivariate analysis, elderly and pharmacist intervention were significant associated factors with bleeding (OR = 2.29, 95% CI: 1.13–4.65 and OR = 0.51, 95% CI: 0.30–0.87, respectively).

**Table 3:** Logistic regression analysis according to Bleeding Academic Research Consortium bleeding greater than type 2

	Univariate analysis		Multivariate analysis	
	OR (95% CI)	*P*-values	OR (95% CI)	*P*-values
Elderly (> 65 years old)^a^	2.57 (1.30–5.07)	0.006	2.29 (1.13–4.65)	0.022
Male	0.89 (0.54–1.46)	0.65		
SCr (> 2.26 mg/dL)^a^	1.01 (0.20–5.11)	0.99		
eGFR (< 60 mL/min/1.73m^2^)	1.68 (1.04–2.70)	0.034	1.44 (0.87–2.37)	0.16
Liver function^a^ (AST > 90 IU/L or ALT > 69 IU/L)	0.50 (0.06–4.22)	0.53	0.78 (0.09–6.80)	0.82
More than two antithrombotic drugs^a^	0.99 (0.52–1.90)	0.97	0.94 (0.48–1.84)	0.86
Pharmacist intervention	0.53 (0.32–0.90)	0.018	0.51 (0.30–0.87)	0.014

Elderly, each laboratory test value, and the number of antithrombotic drugs were the baseline values at the start of observation

*OR* Odds ratio, *CI* Confidence interval, *SCr* Serum creatinine value, *eGFR* Estimated glomerular filtration rate, *AST* Aspartate aminotransferase, *ALT* Alanine aminotransferase

^a^Risk factors included in HAS-BLED (Hypertension, Abnormal liver/renal function, Stroke history, Bleeding history or predisposition, Labile INR, Elderly, Drug/alcohol usage) score

### Cumulative incidence rate of BARC bleeding greater than type 2

**Fig. 3:**
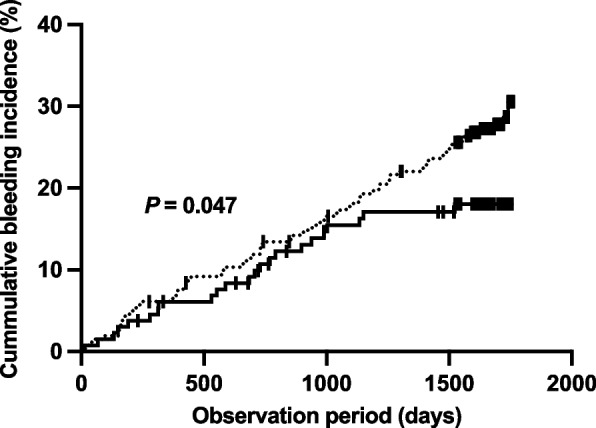
Cumulative bleeding incidence rate. Time-to-event analysis was performed using Kaplan–Meier curves. The dotted line represents the patients who never receive pharmacist intervention, and the solid line represents patients who received pharmacist intervention. The *P*-value was calculated using the log-rank test

Figure 3 shows the Kaplan–Meier curves of the cumulative incidence of BARC bleeding greater than type 2 during the observation period. A significant difference in the cumulative incidence of bleeding between the pharmacist intervention and non-intervention groups was observed (*P* = 0.047). The observation period differed depending on the time of entry, and the numbers of patients at 1500 days were 101 and 190 in the intervention and non-intervention groups, respectively.

### Prescription change

Table 4 shows the description of prescription changes during January–December 2017 in the pharmacist intervention and non-intervention groups. The prescription change rate for all medication and antithrombotic drugs was significantly higher in the pharmacist intervention group compared with the non-intervention group (all medication: 71.2% *versus* 42.0%, *P* < 0.001, antithrombotic drugs: 31.8% *versus* 17.0%, *P* = 0.001). Total prescription change rates of warfarin were 16.7% and 12.1% in the intervention and non-intervention groups, respectively. Most of the changes in the warfarin description were dose adjustments. Total prescription change rates of DOAC were 4.5% and 3.0% in the pharmacist intervention and non-intervention groups, respectively. Total prescription change rates of antiplatelet were significantly higher in the pharmacist intervention group compared with the non-intervention group (10.6% *versus* 1.9%, *P* < 0.001). The discontinuation rate of antiplatelet was significantly higher in the pharmacist intervention group compared to the non-intervention group (*P* = 0.001).

**Table 4:** Prescription changes in the pharmacist intervention and non-intervention groups during January–December 2017

		Intervention group (*n* = 132)	Non-intervention group (*n* = 264)	*P*-values
Prescription changes for all medications		94 (71.2%)	111 (42.0%)	< 0.001
Prescription changes for antithrombotic drugs		42 (31.8%)	45 (17.0%)	0.001
Warfarin	Total changes	22 (16.7%)	32 (12.1%)	0.22
	Dose adjustments	21 (15.9%)	32 (12.1%)	0.35
	Discontinuations	1 (0.8%)	0	0.33
	Drug changes	0	0	1.00
	Initiations	0	0	1.00
DOAC (apixaban, dabigatran, edoxaban, rivaroxaban)	Total changes	6 (4.5%)	8 (3.0%)	0.56
	Dose adjustments	1 (0.7%)	1 (0.4%)	1.00
	Discontinuations	1 (0.7%)	1 (0.4%)	1.00
	Drug change	4 (3.0%)	5 (1.9%)	0.49
	Initiation	0	1 (0.4%)	1.00
Antiplatelet (aspirin, clopidogrel, prasugrel, cilostazole, etc.)	Total changes	14 (10.6%)	5 (1.9%)	< 0.001
	Dose adjustment	0	0	1.00
	Discontinuations	11 (8.3%)	4 (1.5%)	0.001
	Drug change	3 (2.3%)	1 (0.4%)	0.11
	Initiation	0	0	1.00

*DOAC* Direct oral anticoagulant

## Discussion

This is the first report which shows that pharmacist intervention can reduce the risk of bleeding in cardiology outpatients by conducting patient interviews and advising prescription interventions in accordance with various guidelines for anticoagulant therapy.

The rate of bleeding defined as BARC criterion greater than type 2 were 17.4% and 28.4% in the pharmacist intervention and non-intervention groups, respectively, indicating a significant 39% lower bleeding rate by pharmacist intervention. For patients that received antithrombotic drugs during the observed period, the pharmacist recommended the physician to prescribe a shorter duration of multiple antithrombotic drugs and to change a single antithrombotic drug during the maintenance phase. In the WOEST study, the rates of the patients who haemorrhaged as defined by BARC as type 2 and 3 were 31.7% and 14.3% in the triple and double therapy groups, respectively [3], indicating a 55% reduction in bleeding rate by changing the antithrombotic therapy. The AFIRE trial showed that the rate of major bleeding, as defined by the criteria of the International Society on Thrombosis and Haemostasis, was 5.75% in the rivaroxaban plus antiplatelet drug and 4.14% in the rivaroxaban monotherapy [15–17], indicating a 28% reduction in the bleeding rate could be expected by switching to monotherapy during the early phase. Thus, the frequency of bleeding could be reduced by up to 28–55% by facilitating the switch according to the therapy guidelines. The reduction of bleeding rate in the pharmacist intervention group in the present study was comparable to the previously reported values. Furthermore, the pharmacist intervention in this study included not only switching to monotherapy in the early stages, but also dose reduction of anticoagulant drugs based on drug-drug interactions, discontinuation of antiplatelet drugs without appropriate indication, dose reduction due to reducing renal function, and changes to an antiplatelet drug with milder bleeding risk based on the incidence of subcutaneous bleeding. These collaborative medication adjustments with physicians likely contributed in part to the reduction in bleeding events.

Our analysis showed that both elderly and pharmacist non-intervention were significant bleeding risk factors, while renal failure, liver dysfunction and the use of multiple antithrombotic drugs at the initiation of observation did not show an association with bleeding, despite that they were included in the evaluation of the HAS-BLED score. The pharmacist intervention group had a higher ratio of prescription changes for antithrombotic drugs in this study, which might directly affect the reduced risk of bleeding because the use of multiple antithrombotic drugs at the initiation of observation was not associated with the bleeding risk. Renal failure in the HAS-BLED score was defined as a serum creatinine level greater than 2.26 mg/dL, which is present in patients with end-stage renal failure such as haemodialysis [18]. Eight (2.0%, data not shown) and five patients (1.3%, data not shown) among the total patients in this study had renal failure and liver dysfunction, respectively, and renal failure and liver dysfunction were not detected as bleeding risk factors due to the small number of applicable patients. Hypertension and concomitant alcohol intake were not assessed in this study. Although labile INR was also not evaluated in this study, the number of prescription changes of warfarin tended to be greater in the pharmacist intervention group. Changing the dose or discontinuation of warfarin may lead to better control of INR within the optimal range, which may affect bleeding events.

This study found no effect of pharmacist intervention on the mortality of eligible patients. A systematic review reported that pharmacist interventions in patients with heart failure reduced the heart failure hospitalisation rates, but had no effect on reducing mortality [19]. Furthermore, pharmacist intervention as part of a multidisciplinary team reduced the hospitalisation rates, but the pharmacist-only intervention did not lead to a reduction in the hospitalisation rates because the pharmacist-only intervention did not achieve the complete therapeutic management and education of the patient [19]. Since the pharmacist intervention evaluated in our study was not a multidisciplinary team-based intervention, the pharmaceutical outpatient clinic only could not reduce the rate of re-hospitalisation for heart failure. Although it is challenging to intervene with a medical team in an outpatient setting, comprehensive care in the community is important to improve patient outcomes, and future development in this area is expected.

Various intervention methods by pharmacists have been reported [20]. In our study, the pharmacist intervention was conducted by attending the physician consultations in the outpatient clinic. Potential obstacles to pharmacist-physician collaboration were reported as limited trust-building associated with less direct communication, limited communication methods for prescription suggestions and limited discussion time [20]. Previous reports demonstrated that the rate of prescription change was higher in the group where prescription issues were discussed directly between the physician and pharmacist in conference than in the group where the issues were discussed only using written communication [21]. Moreover, providing pharmaceutical information and instruction directly to the patients in a short period of time by the community pharmacists was reported to result in a decrease in HbA1c and improvement in systolic blood pressure [22]. Our pharmacist interviewed patients to check their adherence and the status of adverse events before the physician consultations and intervened in their prescriptions in collaboration with the physician as well as an educational approach for patients at high risk of bleeding [23], which may have led to the improvement of their bleeding event risk. In addition, although the Kaplan–Meier curves of the two groups were similar in the early part of the observation period, the pharmacist intervention group had a lower incidence rate trend in the later part (Fig. 3). The Japanese Circulation Society published a guideline focused on the update antithrombotic therapy in patients with coronary artery disease in 2020 [8] following the results of the AFIRE trial [17], near the third year of the present study observation period. This guideline had a major impact on the antithrombotic therapy including aggressively shortening the duration of multiple concomitant anticoagulation drugs. The pharmacist who provided the intervention in this study suggested both short-term multiple combination therapy and switching monotherapy as possible, considering the risk of bleeding and thrombosis. This change in clinical recommendations can be a turning point in significant changes in clinical outcomes. In addition, the pharmacist carefully adjusted the dosage of antithrombotic drugs according to the long-term fluctuations in renal function during the therapeutic period. The median of the pharmacist intervention period was 224 days, therefore, the interventions described above might result in long-term improvements in clinical outcomes corresponding to the result of the cumulative incidence of bleeding.

There are some limitations in this study. First, this is a small-scale, single-centre, retrospective study and the bias of the institution and pharmacist assigned to the study cannot be eliminated because the intervention was performed by a single specific pharmacist. The results of this study may have been influenced by the experience level of the pharmacist, because this pharmacist had many years of experience in charge of cardiology wards and had professional expertise in this field. And, the statistical power of the post-hoc analysis for the primary end-point was 62.7%. Therefore, the results of this study cannot be directly generalised to all clinical setting for pharmaceutical outpatient clinic of cardiovascular internal medicine. Second, bleeding events defined as BARC was determined from the descriptions in the electronic medical record by researchers. Prospective intervention studies are needed to verify these results. Third, events that occurred outside of the hospital may not have been fully evaluated. Outcomes for patients who no longer visited the hospital during the observation period were evaluated by the report records obtained from the external institutions. Forth, the number and duration of pharmacist intervention varied in this study, and the prescription changes in this study were only evaluated during the observation period in 2017, when the pharmacist intervention for outpatients started.

## Conclusions

In conclusion, pharmacist intervention in the cardiology outpatient clinic can improve the clinical outcomes by reducing the rate of bleeding in patients using antithrombotic drugs, probably because of prescription changes for warfarin dose adjustments and discontinuation of antiplatelet drugs. Future prospective refined intervention studies may demonstrate the further benefits of pharmacist intervention.

## Data Availability

The datasets generated during and/or analysed during the current study are available from the corresponding author on reasonable request.
